# Preconception Care to Reduce the Risks of Overweight and Obesity in Women of Reproductive Age: An Integrative Review

**DOI:** 10.3390/ijerph18094582

**Published:** 2021-04-26

**Authors:** EunSeok Cha, Michael J. Smart, Betty J. Braxter, Melissa Spezia Faulkner

**Affiliations:** 1 College of Nursing, Chungnam National University, Daejeon 305-764, Korea; echa5@cnu.ac.kr; 2 Nell Hodgson Woodruff School of Nursing, Emory University, Atlanta, GA 30322, USA; 3 Byrdine F. Lewis College of Nursing and Health Professions, Georgia State University, Atlanta, GA 30303, USA; mjs.30309@gmail.com; 4 School of Nursing, University of Pittsburgh, Pittsburgh, PA 15260, USA; bjbst32@pitt.edu

**Keywords:** female, overweight, obesity, preconception care, counseling, lifestyle, family planning

## Abstract

Despite adverse pregnancy outcomes for women with overweight or obesity, preconception guidelines for achieving optimal wellness for women contemplating pregnancy regarding the risks of overweight or obesity are varied based upon national affiliation. The aim of this study was to synthesize the best evidence related to preconception counseling and care focused on overweight or obesity provided to women of reproductive age. An integrative review of original studies was conducted. PubMed, Cumulative Index in Nursing and Allied Health Literature, Ovid, Scopus, Web of Science, and Embase were included. Full-text, data-based articles were searched from 2009 to 2018, with reviews and synthesis completed in 2019 and 2020. Of 8703 initial articles, 31 articles remained in the review. Quality assessment and level of evidence were evaluated based upon criteria from the Joanna Briggs Institute and the Johns Hopkins Nursing Evidence-Based Practice Quality Guide. The level of evidence for the majority of studies was non-experimental but they were of good quality with appropriate methods, samples and relevant results. Limited attention and interest in preconception counseling regarding risks of overweight or obesity by health care professionals were noted, which may contribute to women’s unawareness of these risks on preconception health.

## 1. Introduction

Adequate preconception counseling is important for women who are overweight or obese and are planning to become pregnant. Excess weight in the preconception period can influence fecundability (average per-cycle probability of conception), as well as the trajectory of metabolic changes during pregnancy and in the postpartum period due to inflammation, dyslipidemia and insulin resistance [1,2,3]. Overweight and obesity in the preconception period increase the risk of metabolic dysfunction, causing alterations in placental function, fetal vascular formation and gene expression during pregnancy [4,5]. Since there is an escalation of overweight and obesity in women of reproductive age, as well as the effects of delaying pregnancy until later in life, more women become vulnerable to obesity-related pregnancy complications and chronic disorders after the birth such as hypertensive disorders, impaired glucose tolerance and type 2 diabetes [6,7,8]. 

Metabolic processes (e.g., basal metabolic rate, protein usage and storage, triglyceride synthesis, hemodynamic changes) become altered during pregnancy to provide an optimal supply of glucose and protein to mothers and fetuses [1]. These alterations may extend to the 4th trimester (12 weeks post-delivery) or later in life with increases in the risks for complications, such as maternal stroke or delivery of macrosomic infants [9]. A continuum of care from preconception to the 4th trimester for women who are overweight or obese is essential [10,11]. Despite the known risks related to excess weight in women throughout preconception, during pregnancy and the postpartum period, little is known regarding current preconception counseling focused on overweight and obesity provided to women of reproductive age. According to the only systematic review retrieved on preconception intervention programs for improving pregnancy outcomes in women who are overweight or obese, no RCTs are available, limiting the best practice evidence for this growing population [12].

The number of pregnant women who are overweight or obese has increased in both high and middle income countries based upon country-level data collected from the World Health Organization, the World Bank and the Food and Agricultural Organization [8]. For example, in the United States, 41.2% of women aged 20 years old and older were obese in 2015–2016 [13] and about half of women (48%) start their pregnancy being overweight or obese [14]. In response to the obesity epidemic in childbearing women, the Centers for Disease Control and Prevention (CDC) and the Institute of Medicine (IOM) in the United States, and the National Institute for Health and Care Excellence (NICE) in the United Kingdom have focused on overweight and obesity in preconception health since 2006 [15,16,17,18]. The NICE recommends that women should be counseled to have a body mass index (BMI) of less than 25.0 kg/m^2^ when starting a pregnancy, and the Royal Australia College of General Practitioners (RACGP) and the CDC also indicate the importance of preconception care to achieve a healthy weight and lifestyle at the first prenatal visit [19,20]. Newborns of women who are obese are at a high risk for developing neural tube defects [21,22]. As a preventive measure, taking a folic acid supplement is recommended during the childbearing period, although a specific dosage for women with excess weight varies depending on national affiliation [6,20]. The Royal Australian and New Zealand College of Obstetricians and Gynaecolgists (RANZCOG), Royal College of Obstetricians and Gynaecologists (RCOG) and the Joint Centre for Maternal and Child Enquiries (CMACE) in the United Kingdom recommend increasing supplementation of folic acid to 5 mg instead of 400 mcg. The Institute of Obstetricians and Gynaecologists (IOG) in Ireland concurs with the recommendation of the RCOG and CMACE. The American College of Obstetricians and Gynecologists (ACOG) and the CDC, however, do not recommend doses of folic acid greater than 400 mcg, stating evidence does not support a greater dose [6,20].

Studies show that women who are overweight or obese have lower levels of vitamin D and calcium, which are associated with fetal growth, maternal depressive moods, and maternal and fetal bone formation requirements [23,24,25]. Thus, the RCOG and CMACE in the United Kingdom and the IOG in Ireland, but not the ACOG, include these micronutrients in preconception counseling, although recommendations for sufficient and safe amounts for women who are overweight or obese remain unanswered [6,25]. Similarly, RANZCOG recommends daily supplementation of iodine that is not included in other guidelines, including ACOG.

Systematic reviews have shown there are missed opportunities to provide lifestyle interventions to address pregnancy risks in women who are overweight or obese, as well as those who may be prone to excess weight gain in the preconception period and pregnancy [26,27,28]. A healthy, balanced, diet without excessive caloric intake during the preconception period is important to prevent teratogenesis and gestational diabetes and to generate normal fetal programming of the adrenal–pituitary–hypothalamic axis during gestation [11]. Regular physical activity in the preconception period may lead to gestational diabetes prevention, improved fecundability, and motivation to continuously exercise during pregnancy [12,28,29]. At least one-BMI-unit weight loss or 10% or more of weight loss may be beneficial to improve fecundability and reduce adverse pregnancy outcomes in women [11,30].

Despite the potential benefits of preconception health interventions focusing on the effects of overweight or obesity in women of reproductive age, current preconception interventions delivered in public health and community settings are mostly focused on general health topics such as drinking cessation, taking multivitamin or folic acid supplements, and immunizations, rather than on obesity risk [31,32]. Furthermore, interventions to improve maternal and fetal cardiometabolic health are usually initiated after conception and results are often unsuccessful [32,33]. There is a great need for obesity-specific preconception counseling to reduce risk associated with excess weight prior to and during pregnancy.

The aim of this study was to synthesize the best evidence related to preconception counseling and care focused on the effects of overweight or obesity provided to women of reproductive age.

## 2. Materials and Methods

### 2.1. Design

An integrative literature review was conducted using the process as described by Whittemore and Knafl that allows for the combination and synthesis of both non-experimental and experimental design studies [34]. This process includes clarity in problem identification, literature review, evaluation of the data from empirical reports, data analysis from primary resources, and the final presentation of the summary of the findings that have pragmatic relevance, particularly for a practice discipline [34]. The Preferred Reporting Items for Systematic Reviews and Meta-Analyses (PRISMA) guidelines were incorporated for presenting information retrieved from the integrated review [35].

### 2.2. Search Methods

The electronic databases PubMed, Cumulative Index in Nursing and Allied Health Literature (CINAHL), Ovid, Scopus, Web of Science, and Embase were used for the search. Articles were retrieved that were published over a ten-year period from 2009 to 2018 with article reviews occurring in 2019 and final synthesis in 2020. A combination of search terminologies related to the integrative review topic was used (i.e., women of reproductive age; young women; childbearing women), preconception, education (i.e., counseling; education; intervention), and lifestyle (i.e., lifestyle modification; lifestyle intervention; healthy lifestyle; diet; physical activity; exercise; weight loss). To conduct a comprehensive review of the literature on preconception counseling, the terms of overweight or obesity were not included in the search terminology to prevent the inclusion of studies that included *only* women with overweight or obesity. Rather, the investigators manually reviewed each article during the title, abstract and full-text review steps to check the eligibility of studies that addressed overweight or obesity prior to pregnancy.

### 2.3. Inclusion and Exclusion Criteria

The inclusion criteria for target studies were publications that addressed preconception counseling or care in women of reproductive age and were full-text, data-based articles written in English. Due to the global epidemic of obesity, studies completed both within and outside of the U.S. were included. The exclusion criteria were: (1) abstracts or conference proceedings; or (2) articles that focused on preconception counseling and care for women with preexisting diseases (e.g., cardiac problems, diabetes, and other chronic conditions). We also conducted ancestry searches of the reference lists of the included articles to find other articles for inclusion in this review. The composite search revealed a total of 8703 potential articles for review.

**Figure 1:**
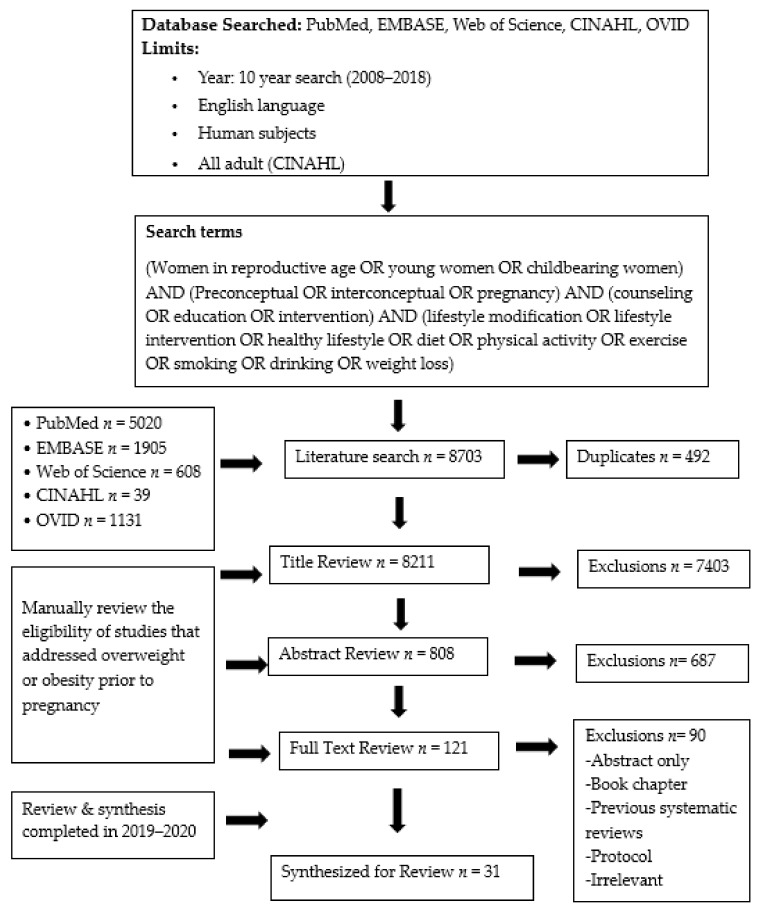
PRISMA (The Preferred Reporting Items for Systematic Reviews and Meta-Analyses) Flowchart to select articles reviewed.

Following the search of the selected databases, teams of investigators (3 doctoral-prepared nurse researchers and 1 graduate nursing student) at 2 separate universities began the process of reviewing articles. The complete dataset of articles was split between the two respective sites. The initial screen of the articles included an examination of the title for appropriateness of content, followed by a review of abstracts and finally, a full-text review of the articles. Any duplication of articles was eliminated in the review process. Figure 1 presents the PRISMA flow diagram, numbers of the articles obtained and those excluded in each step of the review, and the final 31 full-text data-based original articles included for the synthesis of findings.

### 2.4. Quality Appraisal

Quality assessment and grading of evidence was completed in a 2-step process. The quality of each article was determined by scoring using the appraisal checklists for either quasi-experimental studies (non-randomized) or randomized controlled trials as appropriate based upon the Joanna Briggs Institute (https://jbi.global/critical-appraisal-tools (accessed on 9 February 2019). The authors randomly selected articles that one another reviewed to check the thoroughness and quality for each other and to determine inter-rater consistency for inclusion of articles for full review. The level of evidence for grading the published studies in the full-text review was confirmed by two investigators based upon the Johns Hopkins Nursing Evidence-Based Practice Quality Guide from Level I (randomized controlled trials), Level II (quasi-experimental studies), Level III (non-experimental studies; quasi-experimental and non-experimental studies), Level IV (clinical practice guidelines; position statements) to Level V (integrative reviews, case reports, experiential evidence of national experts) [36]. Quality rating was indicated as high, good or low depending on the adequacy of the study designs, sample sizes and appropriateness of study conclusions. Descriptions of the ratings are as follows:

A. High quality: Consistent, generalizable results; sufficient sample size; adequate control; definitive conclusions; consistent recommendations based on comprehensive literature review that includes thorough reference to scientific evidence.
B. Good quality: Reasonably consistent results; sufficient sample size; some control, fairly definitive conclusions; consistent recommendations based on fairly comprehensive literature review that includes some reference to scientific evidence.
C. Low quality or major flaws: Little evidence with inconsistent results; insufficient sample size; conclusions cannot be drawn [36].

### 2.5. Data Abstraction

The 31 articles were grouped based upon the following content areas that emerged during synthesis of the findings and major categories of themes that align with the study purpose. The categories include the following: (1) knowledge of preconception health in women of reproductive age; (2) lifestyle and weight management behaviors and potential impact on maternal and neonatal health; (3) preconception counseling provided by health care professionals; and (4) preconception educational interventions for childbearing women. The categories are not mutually exclusive since some studies included findings relative to more than one category. 

### 2.6. Synthesis

Once articles were identified for the final review, a doctoral-prepared nurse researcher and the PhD student at one-site rechecked the eligibility of the articles selected. A nurse researcher and the student each independently reviewed the study purpose, quality and findings of each study to identify a thematic category that aligned with the purpose of this review. Once themes were identified with associated studies, a second nurse researcher reviewed and validated the synthesis of the thematic categories.

## 3. Results

The results of the data abstraction and findings related to the major categories of themes in the data are described in the following section. A summary of information from full-text articles is included in Table 1.

### 3.1. Knowledge of Preconception Health in Women of Reproductive Age

Childbearing women’s awareness and knowledge of preconception health were examined by seven studies [29,37,38,39,40,41,42] and women’s perception of body size was examined by two studies [38,43]. Participants in these studies were recruited from clinics or primary care settings [38,39,40,42,43] and internet-based platforms [37,41], while one study reviewed the information accuracy of preconception health in websites using search terms developed by interviews with women and health care professionals [29].

Overall, women’s preconception health knowledge on folic acid supplementation, vaccination, mother’s age for positive health outcomes, benefits of maintaining a normal weight, smoking cessation, sexually transmitted infection screening and avoidance of substance use was high [37,39,40,41,42], and knowledge regarding the risk in obesity and avoiding high-caloric food items during pregnancy was low to moderate [29,38,39,41,42]. Many women were not aware of the benefits of physical activity during pregnancy and considered regular exercise as potentially harmful to their unborn baby [42].

Women who were overweight inaccurately perceived their body size and/or rarely understood the impact of weight on pregnancy outcomes [38,41,43]. Women with obesity versus overweight had higher intentions to lose weight [43], and the intention of overweight women to engage in behavioral changes was not high (only 53% women intended to make lifestyle changes) [42].

In research on the use of the internet to obtain information on preconception, women with excess weight sought information on “how to get pregnant”, rather than how to have a “healthy” pregnancy [29]. Risk related to obesity was rarely selected during internet searches by the women which may indicate the unawareness of the obesity-related pregnancy risk. The most common searches were folic acid supplementation, rubella vaccination, smoking cessation, preconception counseling in general by health care professionals, and doctor’s advice for medication, while topics related to managing weight such as doing regular exercise were rarely included [29].

Women were not aware of and did not accurately evaluate their overweight and obesity risks on reproductive health [43]. Their perceived health was viewed mostly as good or very good, and consulting with health care providers to lose weight was often interpreted as being rude, accusing and unrealistic advice [43,44]. The time to start to worry about health was usually noted to occur after conception [45]. While women were informed about what is needed for an optimal pregnancy experience and delivery of a healthy infant (e.g., avoidance of excessive weight gain during pregnancy and alcohol consumption, taking folic acid supplements, and healthy nutrition), they reported a lack of understanding of the reasons, which led to poor adherence [43,45].

### 3.2. Lifestyle and Weight Management Behaviors and Potential Impact on Maternal and Neonatal Health

Six studies examined healthy lifestyle and weight management behaviors including diet, exercise, and weight loss strategies in women of childbearing age [43,46,47,48,49,50]. Women who wanted to become pregnant practiced diverse weight management strategies, although some of them were unhealthy [47]. Examples of unhealthy behaviors were smoking, fast food and soda consumption and fewer servings of vegetables [47]. Weight loss strategies were taking diet pills, diuretics, and vomiting and fasting for 24 h or more. Confidence in losing weight was low among women who were overweight or obese [43].

Women did exercise, although the majority of women (49.6–78.7%) did not meet current exercise recommendations of 30 min of exercise per day, five days per week [43,46,49]. Intention to adhere to exercise recommendations was low, especially in women who were overweight compared to those with normal weight or obese [43]. Likewise, a poor rate of adherence to following dietary recommendations was also found [46,47,50]. According to Berenson et al. [47], many women did not meet the daily recommended intake of fruits and vegetables: 76% consumed less than one serving of fruit, 90% consumed less than one serving of green salad, and 72.9% consumed less than one serving of vegetables. Similarly, Inskip et al. [46] found that about 53% of participants did not comply with the recommended intake of fruits and vegetables. Women with obesity showed a tendency to consume high amounts of meats and sweets, which are risk factors of developing gestational diabetes (RR of 2.08; 95% CI = 1.18−3.62) [50].

Maternal weight status and lifestyle behaviors in the preconception period influenced maternal and neonatal health according to the findings of four studies. These included online surveys [51] and national prospective cohorts [52,53,54]. Fecundability was decreased in women with BMI ≥35 or women with a waist circumference above 36 inches or who had gained ≥40 pounds since the age of 17 years [51]. Physical activity, especially vigorous physical activity during the preconception period, increased fecundability in women who were overweight or obese [51]. However, it did not prevent pre-eclampsia [53] or delivery of abnormal birth weight infants [52].

Both the time of developing overweight or obesity in women and their race were found to have effects on neonatal health [52]. While being adolescent mothers who were overweight or obese during pregnancy increased the risk for having infants with low birth weight (OR of 3.89; 95% CI = 1.26–12.04, with the OR increasing after adjusting for confounders and prenatal mediators to 4.59; 95% CI = 1.26–16.70), adult women with overweight or obesity demonstrated an increased risk for infant macrosomia (OR of 1.56; 95% CI = 1.02–2.38). A later study by Strutz et al. [54] showed a moderation effect by race. African American women that were overweight or obese had a higher risk of delivering an infant with macrosomia (OR of 3.83; 95% CI = 1.02–14.36) compared to other racial/ethnic cohorts.

### 3.3. Preconception Counseling Provided by Health Care

#### Professionals

There were seven studies that examined the effects of preconception counseling by health care professionals on women’s health and behaviors [15,17,18,40,45,48,55]. Preconception counseling encouraged women to undertake preventive and proactive health behaviors in order to have a healthy pregnancy (e.g., daily consumption of multivitamins prior to conception, first-trimester entry into prenatal care). However, benefits were not always evident in behaviors such as diet and exercise in women who were overweight or obese [17,18,40,48]. Potential reasons for this were the passive attitudes of health care providers to deliver preconception counseling and their lack of capability to provide client-centered counseling [40,45].

Less than 20% of women reported they received a health care provider’s advice on healthy behaviors during the preconception period [55]. Although counseling regarding smoking or drinking was offered more to women who were overweight or obese compared to normal weight or underweight women, the content was not obesity-specific and advice on maintaining a healthy BMI occurred more during pregnancy, not in the preconception period [15]. Women were skeptical about advice to lose weight to improve preconception health and had low confidence in changing their lifestyle during the preconception period. Additionally, health care professionals did not play an active role in changing women’s perception and behaviors [40,43,45], despite an unmet need for ways to obtain a healthy weight and maintain a physically active lifestyle.

Health care providers often reported time restrictions, low policy priority, and a “not my responsibility” attitude to providing preconception counseling [43,45,55]. General practitioners reported that preconception care was not their practice area and that it should be a targeted public health campaign or delivered by a midwife or nurse practitioner. They indicated that peer education would be better than education by health care providers [45,55]. Outsourcing education programs such as community programs, online education, or mobile applications that focus on diet and exercise were listed by health care professionals as resources they wanted to recommend to clients that need to lose weight [43]. Although there were concerns about general practitioners providing preconception care since they may lack information in reproductive health [45], women visiting an OB-GYN were found to be less likely to receive diet and exercise counseling compared to women visiting a non-OB-GYN provider [17].

About two-thirds of women reported positive feedback on receiving guidance from their health care professionals that specifically addressed pregnancy-related risk regarding alcohol consumption, tobacco use, taking certain medications, and obtaining HIV testing [40,48]. However, in other research, about half of women (49%) reported no preconception health advice was given by their health care providers [55]. Due to limited chances to obtain preconception health advice from health care professionals, women used the internet, friends and family, books and media to obtain information related to preconception health [44,55].

Childbearing women showed a desire for preconception counseling and care from health care professionals, regardless of culture, age or weight status [43,44,45]. More women (75%) wanted to receive preconception care in the future and two-thirds of them wanted to have multiple counseling sessions [44]. However, general advice on healthy lifestyle behaviors (e.g., diet or exercise) did not influence behavioral change in women [48,55]. Unfortunately, due to a lack of awareness of the importance of preconception health, obtaining information on this topic remains a low priority for some women. A dismissive attitude of health care professionals has contributed to a diminished focus on preconception health for childbearing women with overweight or obesity [18].

### 3.4. Preconception Educational Interventions for Childbearing Women

Nine studies examined the effects of interventions on preconception education [29,39,56,57,58,59,60,61,62]. Changes in knowledge of preconception health, self-efficacy on behavioral change and actual behavioral change were evaluated in response to varied intervention approaches: individualized education interventions [37,39,56,57,58,60,61,62], group interventions [59] and eHealth interventions [37,61]. Despite the acknowledgement of obesity’s risk on having a healthy pregnancy, some studies did not include obesity-related measures as preconception intervention outcomes [39,57,61]. 

Most interventions were focused on general preconception education such as taking folic acid supplementation and alcohol cessation [37,39,57,58,59,61]. Approximately half of the studies were focused on the reduction of obesity risks to have a healthy pregnancy [37,56,60,61]. Only two studies emphasized the significance of a healthy lifestyle in women planning to become pregnant [59,62].

Increased preconception knowledge was associated with actual behavior changes in some studies. Once preconception knowledge was improved, improved behavioral changes were found in folic acid consumption, vaccinations, smoking and drinking, and antenatal check-ups [37,56].

Interventions on weight loss during the preconception period revealed different methods and inconsistent findings [37,56,59,60,62]. For instance, women who were overweight or obese experiencing gestational diabetes in their previous pregnancy lost their weight after one-year follow-up as the result of a 4-week lifestyle intervention prior to a subsequent pregnancy [60]. Women who received a motivational interviewing (MI) intervention lost more weight than those not receiving the MI component of the intervention prior to fertility treatment (9.3 kg vs. 7.3kg, *p* = 0.01) [63]. In other studies, results were not adequate. Women who were overweight or obese and received interventions about dieting or exercising to lose weight before pregnancy did not show any differences in behavioral or weight changes compared to those who did not [48,62], while the advice on smoking and taking vitamins before pregnancy successfully aided in behavior change [48]. Despite the failure of weight loss through lifestyle modification in a 6-month intervention in the preconception period completed by van Oers et al. [62], the women that did not gain additional weight, compared to those that gained weight, showed lower hypertensive complications.

## 4. Discussion

Screening of metabolic health risks in the preconception period aids in preventing the negative effects of obesity on maternal health and fetal development [28,64,65]. Currently, there are no specific evidence-based clinical guidelines in the US for women who are overweight or obese regarding recommendations for healthy weight, diet and physical activity during the preconception period [6,20,28]. Professional organizations such as the American Diabetes Association (ADA), American College of Obstetricians and Gynecologists (ACOG), American Heart Association (AHA) and Academy of Nutrition and Dietetics Foundation have released recommendations, which do not include adequate obesity-specific pregnancy risks in the preconception period [11,64,66,67]. The lay public can be easily confused and ignore the importance of preconception health and the effect of excess weight prior to conception.

This integrative review adds information on an under-researched health concern regarding what is known about knowledge and interventions focused on obesity-related pregnancy risks during preconception. It provides evidence on the significance of addressing obesity-related pregnancy risk and the benefits of exercise and healthy nutrition. Evidence on risks related to the use of diet pills and diuretics to promote weight loss and the absence of a diet comprised of the recommended amounts of fruits and vegetables in the preconception period supports the importance of including nutritionists or dieticians in preconception counseling. Inconsistent guidelines and deficient knowledge on the value of a healthy lifestyle during preconception, exist due to greater emphasis on women’s interest in seeking information on “how to become pregnant” and not on how to have a “healthy pregnancy”. Current research supports the need for interventions that target specific high-risk groups that experience overweight or obesity, such as during adolescence and those delaying pregnancy.

The level of evidence for the majority of studies in this integrative review was non-experimental in design, but they were of good quality with appropriate methods, samples and relevant results. Although the review process included manual examination of each article to ensure that the study addressed the risks of overweight or obesity prior to pregnancy, there is the possibility that this review was not exhaustive.

Major findings included that preconception physical activity and weight loss decreased the risk for developing gestational diabetes and hypertensive disorders in pregnancy. However, there remains a dearth of randomized, controlled studies to validate effective practice recommendations during preconception [12]. The findings of this review are primarily based on descriptive studies and should be interpreted with caution. Therefore, further investigations are needed to identify the effects of social determinants on preconception health, which are essential to design effective interventions [26,27,54].

## 5. Conclusions

Evidence from this review supports women’s unawareness of the risks related to overweight or obesity prior to pregnancy and the low attention paid to preconception counseling by health care professionals [40,45]. One possible explanation is the minimal emphasis on guidelines or standards of care for women who are overweight or obese and the lack of synthesis of best practices based upon well-controlled interventions for women at risk for gaining excess weight during the preconception period. Nurses, nurse midwives, nurse practitioners, and other primary care providers can take the lead in addressing the priority for establishing guidelines for standards of care for preconception counseling in this area. In particular, the collaboration of nurse researchers in the conduct of well-designed studies to further develop the best evidence to prevent overweight or obesity and address priorities of care for women who have elevated BMI prior to pregnancy is needed. Nurses in advanced practice roles can offer their voices by engaging with national and international organizations focused on women’s health for policy implementation in establishing universal standards of care related to overweight or obesity risks during preconception for ensuring a healthy pregnancy. Strategies also include partnering with nursing and other health leaders who are members of the World Health Organization’s Collaborating Centers for Nursing and Midwifery to unify a shared vision for addressing the epidemic of overweight or obesity on maternal and infant outcomes through coordinated efforts in preconception care.

## Figures and Tables

**Table 1:** Summary of full-text, data-based articles written in English.

Author	Study Location	Purposes and Aims	Study Design	Methods	Participants	Key Findings	Level of Evidence
Agricola [29]	Italy	Evaluate the extent to which website recommendations matched those from the American College of Obstetricians and Gynecologists (ACOG)	Cross-sectional descriptive design	Obtained search terms from women planning to become pregnant and healthcare providers; evaluated the first 10 websites from each search term and compared to ACOG guidelines	Women intending to become pregnant (*n* = 21) and health care providers (*n* = 18)	Information on the internet regarding preconception counseling was only 25% accurate; women did not search for information related to obesity in pregnancy; no data were provided on BMI characteristics of the sample.	IIIB
Agricola [37]	Italy	Evaluate an individualized education intervention	Quasi-experimental design	Evaluated individualized, web-based education intervention and evaluated outcomes related to behaviors and knowledge	Women of childbearing age (*n* = 282)	There was improvement in knowledge related to preconception counseling after the intervention; however, there was low baseline knowledge of the risks associated with pregnancy that did not improve with the intervention; 21.3% of the total sample had a BMI >25 kg/m^2^.	IIB
Beckmann [56]	Australia	Evaluate the effectiveness of a structured preconception care program on maternal/fetal outcomes	Case–control retrospective analysis of structured preconception care program	Participants had at least one 90-min session with a midwife; focused on education, vaccinations, and specialist consultation for high-risk patients	Women intending to become pregnant who participated in an intervention (*n* = 56) and those who did not participate (*n* = 168)	Women who received the intervention reported engaging in healthier behaviors; the intervention group had less weight gain and less hypertensive disorders during pregnancy when compared to the control group; mean BMI during the preconception period was 26.8 kg/m^2^.	IIIB
Berenson [38]	USA	Evaluated obesity risk knowledge, weight perception, and health-related attitudes	Cross-sectional descriptive design	Evaluated obesity risk knowledge, weight perception, and health-related attitudes and compared differences between women who were intending to become pregnant with those without intention using survey	Women of reproductive age and patients of a reproductive health clinic (*n* = 1420)	About half of the women intending to become pregnant had low knowledge related to the risks of obesity; women who were overweight were likely to misperceive their weight (71.3%), and report confusion about health diets (76%); 28% of participants were overweight, and 35.6% were obese.	IIIB
Berenson [47]	USA	Evaluate nutritional habits and weight strategies in reproductive-aged women	Cross-sectional descriptive design	Evaluated nutritional habits and weight loss strategies in women trying to conceive and those not trying using survey	Women of reproductive age and patients of a reproductive health clinic (*n* = 1711)	Women trying to conceive engaged in multiple unhealthy behaviors to lose weight including diet pills, laxatives, fasting for 24 h; 27.5% of participants were overweight and 33.5% were obese.	IIIB
Bortolus [45]	Italy	Evaluate attitudes of Italian women of childbearing age and health care professionals regarding preconception health	Qualitative study (focus group interviews)	Focus group interviews focused on women’s questions about preconception health, barriers to preconception care, and attitudes of both women and healthcare providers towards preconception care	One nulliparous group (*n* = 8); one multiparous group (*n* = 6); and one health care provider group (*n* = 12)	Women did not view weight before conception as a concern and did not try to obtain information related to preconception counseling; healthy behaviors changed upon learning that they were pregnant, not during the preconception period.	IIIB
Bye [15]	United Kingdom	Evaluate recall of pregnant women on healthcare provider preconception counseling of healthy BMI	Cross-sectional descriptive design	Survey in antenatal clinics; women’s recall of healthcare professionals counseling of healthy lifestyle including tobacco and alcohol use and weight management strategies	Women with overweight (*n* = 232), normal weight (*n* = 632), and underweight (*n* = 54) who were currently pregnant	Only 33% of women with an elevated BMI received preconception counseling related to weight; 25% of the sample were overweight or obese.	IIIB
Dunlop and Dretler [57]	USA	Evaluated reproductive risks and acceptability of preconception counseling intervention in a low-income clinic	Exploratory sequential design; Used mixed methods survey and a focus group interview	Participants filled out a preconception risk questionnaire then had a brief, individualized intervention based on risks; qualitative interviews were performed with all participants to evaluate the intervention	African American women of reproductive age (*n* = 150)	Preconception counseling was well received from participants; the low-income WIC clinic was found to be an appropriate site to identify women with preconception risk; obesity characteristics of the sample were available.	IIIB
Dunlop and Logue [39]	USA	Evaluate preconception knowledge and related risks and outcomes from brief education intervention	Quasi-Experimental Study	Participants in the intervention group received a brief, 10-min individualized education intervention on specific risk factors; the control group did not receive a specific intervention but their providers were educated about the benefits of preconception counseling; knowledge was assessed between 3 and 6 months after intervention	Low-income African American and Hispanic women of reproductive age; intervention group (*n* = 281) and control group (*n* = 263)	The intervention resulted in improvement of knowledge related to preconception health compared to the control group; knowledge related to obesity was not evaluated or discussed; obesity characteristics of the sample were not discussed.	IIIB
Elsinga [58]	Netherlands	Assess preconception education intervention and outcomes	Quasi-experimental study	Performed knowledge pretest, education intervention, and then measured outcomes including knowledge, behavior change, and maternal/fetal outcomes	The intervention group included *n* = 211 participants; controls were matched in 2:1 ratio by chart review (*n* = 422)	The intervention resulted in improved knowledge in the intervention group, including knowledge related to obesity; obesity characteristics of the sample were not discussed.	IIIC
Goossens [44]	Netherlands	Evaluate women’s needs and preferences regarding preconception care	Cross-sectional descriptive design	Assessed knowledge and needs assessment in reproductive age women that included many topics including obesity, nutrition, environmental exposures, and supplements	Reproductive-aged women intending to become pregnant (*n* = 242)	About half of participants were interested in receiving preconception counseling; women with obesity reported a greater need for lifestyle support than women who were not obese (81% vs. 19%); 19% of participants were overweight and 4% were obese.	IIIB
Harden [43]	USA	Evaluate perceptions of preconception weight-loss interventions in both patients and providers	Mixed methods (cross-sectional survey and a focus group interview)	Cross-sectional study evaluating patients’ willingness to attend weight loss interventions as well as providers’ willingness to refer. Focus group afterward included three women with obesity to further explore weight-loss interventions and intentions	Participants (*n* = 63) included women with or without intention to become pregnant and included both women with normal weight and overweight; providers included MDs, residents, and APPs (*n* = 21)	Patients were interested in preconception weight management interventions; 45.9% of participants underestimated weight status; 57% were able to cite the correct recommendations for physical activity; 25.4% of the sample was overweight and 46.2% was obese.	IIIB
Harelick [40]	USA	Evaluate health behaviors, knowledge of risk factors, and recommendations of healthcare providers	Cross-sectional descriptive design	Evaluated risk factors, knowledge, and recommendations of health care providers regarding preconception health	Convenience sample of reproductive-aged women at a community care clinic (*n* = 340)	Women had good knowledge of risk factors; two-thirds of participants recalled being counseled about preconception risks; 25.4% of participants were obese or overweight.	IIIB
Hillenmeier [59]	USA	Evaluate intervention intended to improve health behaviors and health status of women intending to become pregnant	Randomized control trial	The intervention group included six biweekly group sessions focused on diet, exercise, and vitamin supplementation, substance use, STIs; post-assessment of knowledge was completed 3, 6, and 12 months after the intervention	Reproductive aged women in low income, rural communities; 2:1 ratio of intervention group (*n* = 252) to control group (*n* = 110)	Participants in the intervention group had improved self-efficacy and healthy behavior changes; there was no change in biometric data; BMI data of the participants were not provided.	IB
Hu [60]	China	Evaluate lifestyle intervention program to reduce future risk of GDM in women with a prior history of GDM	Randomized control trial	Intervention group received personalized recommendations from dietician regarding diet and also received personalized exercise recommendations; the control group received generic information on diet and exercise at regular intervals; measured several outcomes including anthropomorphic data, blood pressure, insulin resistance, and other biometric data; also evaluated changes in diet and exercise habits.	Women of reproductive age with a history of gestational diabetes mellitus; included both control group (*n* = 594) and intervention group (*n* = 596)	The intervention group had a significant reduction in weight (1.40 kg vs. 0.21 kg, *p* = 0.001), reduced BMI, waist circumference, improved physical activity behaviors, and reduced insulin levels; 28.2% of participants were overweight and 13.8% were obese.	IB
Inskip [47]	United Kingdom	Examine women’s adherence to nutrition and lifestyle recommendations	Cross-sectional descriptive design	Survey questionnaires were given to women to evaluate diet, physical activity, tobacco use, alcohol use, and nutritional supplement use over the prior three months	Included women of reproductive age who were not pregnant (*n* = 12,445) and who had become pregnant in the prior three months (*n* = 238)	Women who were intending to become pregnant had only slightly healthier behaviors regarding preconception risk when compared to those not intending to become pregnant including smoking, healthy diets, physical activity, alcohol use, folic acid supplementation; obesity characteristics of the sample were not given.	IIIB
Karlsen [63]	Denmark	Evaluate the effect of a motivational interview-based intervention on weight loss in women with overweight or obesity	Retrospective study design	Retrospective study evaluating motivational interviewing-based intervention and evaluated weight-loss outcomes; the intervention group received a mean of 8.6 sessions; both the control and intervention group were given advice related to physical activity and weight loss	Women with obesity who attended an infertility clinic; control group (*n* = 77) and intervention group (*n* = 110)	The intervention group had more weight loss when compared to the control group; all women in the study had a BMI > 30 kg/m^2^.	IIB
McKinnon [51]	Canada and USA	Examine associations between weight, physical activity, and fecundability	Prospective cohort study design	Internet-based prospective cohort study that used questionnaires to assess BMI and distribution of weight gain, physical activity; researchers compared this to self-reported pregnancy and time to pregnancy	Reproductive-aged women ages 21–45 (*n* = 2062)	Obesity and being overweight were associated with decreased fecundability; physical activity was associated with improved fecundability in all women.	IIIB
Oza-Frank [48]	USA	Examine associations between maternal behaviors before and after pregnancy and preconception counseling	Retrospective/secondary analysis using a national dataset	Secondary analysis of Pregnancy Risk Assessment Monitoring System (PRAMS) data in which women answered various internet-based questions related to pregnancy including health behaviors and outcomes. Included questions on folic acid supplementation, alcohol use, tobacco use, and weight-loss strategies	Women who had recent pregnancy in prior 2–6 months (*n* = 3446)	Preconception counseling was not associated with changes in diet, exercise, tobacco, or alcohol use, but was associated with improved folic acid use (OR 2.99, 95% CI 2.24–4.00); 23.6% of those that answered were overweight and 21.0% were obese.	IIIB
Pandolfi [41]	Italy	Evaluate the prevalence of preconception risk factors and related knowledge of adverse pregnancy outcomes	Secondary analysis	Web-based study that evaluated risk factors related to pregnancy including tobacco use, alcohol use, folic acid supplementation, BMI, and maternal disease among other data and compared this to knowledge related to adverse pregnancy outcomes	Reproductive aged women planning a pregnancy within the following year (*n* = 728)	There was limited awareness of the risks that obesity/being overweight have on pregnancy outcomes (34.4%); there was a high prevalence of risk factors for adverse pregnancy outcomes, including alcohol use, tobacco use, folic acid supplementation and vaccine uptake; 21.4% of the sample had a BMI >25 kg/m^2^.	IIIB
Robbins [49]	USA	Evaluate the prevalence of risk factors related to pregnancy	Secondary analysis using two national datasets	Secondary analysis of survey data from two surveillance systems Behavioral Risk Factor Surveillance System (BRFSS) and PRAMS that evaluated multiple self-reported risk factors including depression, diabetes, hypertension, physical activity, and multivitamin supplementation	Reproductive-aged women with recent pregnancy (*n* = 177,314)	Overall prevalence of obesity or being overweight was 44.9%, and varied between different racial groups and among socioeconomic factors; only 50.4% of women met physical activity recommendations.	IIIB
Schoenaker [50]	Australia	Examine associations between pre-pregnancy dietary patterns and incidence of gestational diabetes	Prospective cohort study	Survey of women who were a part of a larger cohort study without a history of diabetes that assessed diet and compared this to self-reported incidence of gestational diabetes	Women born between 1973 and 1978 (*n* = 3853)	A dietary pattern consisting of processed foods including red and processed meats as well as sugar was associated with a higher risk for gestational diabetes mellitus (GDM) that increased with BMI; Mediterranean-style diets were associated with a healthy weight, lower GDM risk, and higher physical activity; 19.5% of the sample were overweight, and 10.5% were obese.	IIIB
Stephenson [55]	United Kingdom	Evaluate the extent to which women prepare for pregnancy	Cross-sectional descriptive design	Evaluation of women’s knowledge, utilization of preconception care, and pregnancy planning using survey questionnaires. Also evaluated related behaviors including alcohol and tobacco use, and vitamin supplementation, among others. Healthcare providers were also interviewed regarding clinical guideline implementation in clinical practice and perceived barriers	Pregnant women who attended maternity services in UK (*n* = 1173); interviews were conducted with healthcare professionals including nurses, physicians, and midwives (*n* = 20)	Only 34% of participants reported receiving preconception counseling regarding unhealthy behaviors; this may have been impacted by the confusion of the provider on who is responsible for preconception counseling; 5% of participants were reported to be obese.	IIIB
Strutz [52]	USA	Assess pre-pregnancy health status and infant birth weight	Longitudinal study design	Survey data of participants of a longitudinal study that evaluated preconception behaviors including obesity, tobacco and alcohol use, and physical activity, among others, and compared these to infant weight	Cohort of young adults aged 18–32 (*n* = 3436) who had singleton live births	Adult-onset obesity was associated with a macrosomic infant; 28% of participants reported low physical activity; 55.6% of participants reported being obese during adolescence and/or adulthood.	IIIB
Strutz [54]	USA	Evaluate preconception risks and impact on birth outcomes and which of these factors impact ethnic disparities in birth outcomes	Secondary analysis of longitudinal study data	Evaluated data from a longitudinal study that evaluated preconception behaviors and as well as maternal/fetal outcomes	Cohort of young adults of reproductive age who identified as non-Hispanic White, non-Hispanic Black, Mexican-origin Latina, or Asian/Pacific Islander with live birth; (*n* = 2292 female respondents; *n* = 3014 live births)	Black females with obesity may be at higher risk of having offspring with low birth weight and macrosomia compared with their normal-weight counterparts; 50.1% of births were associated with maternal obesity during adolescence and/or adulthood.	IIIB
Tyldum [53]	Norway	Evaluate the relationship between physical exercise before pregnancy and risk of pre-eclampsia	Prospective cohort study	Physical activity was assessed through survey data in non-pregnant; subsequent data related to pregnancy and pre-eclampsia were obtained from a national registry	Included women of reproductive age participating in a larger cohort study (*n* = 3489)	There was no increased risk between pre-pregnancy physical activity and pre-eclampsia; 39% of participants were overweight, and 51% were obese.	IIIB
van Dijk [61]	Netherlands	Evaluate the effects of personalized mobile health coaching on health behaviors	Quasi-experimental design	Survey data that evaluated health behaviors of participants who received a 26-week mobile health coaching intervention via text messaging. Evaluated weight, diet, supplement use, and tobacco and alcohol use	Included both men (*n* = 215) and non-pregnant women (*n* = 676) intending to become pregnant that completed the mobile health intervention	The e-Health intervention targeting unhealthy behaviors in the preconception period may help to improve these behaviors and improve fertility; 27.1% of women were overweight and 14.7% were obese.	IIB
Van Oers [62]	Netherlands	Evaluate periconception weight and maternal/fetal outcomes	Randomized clinical trial	Post hoc analysis of both control and intervention groups of a randomized control trial that assessed changes in BMI in women with ongoing pregnancy and evaluated maternal/fetal outcomes including gestational diabetes, preterm birth, hypertension, preterm birth, infant weight, APGAR scores, and perinatal death, among other variables	Women who were obese and treated for infertility (*n* = 244)	There was no significant difference in the prevalence of small-for-gestation (SGA), large-for-gestation (LGA), or adverse neonatal events among women with weight changes during pregnancy; periconceptual weight loss may be associated with less hypertensive complications during pregnancy; all participants included in the study were obese.	IB
Vause [42]	Canada	Evaluate preconception knowledge and attitudes of women at an infertility clinic	Cross-sectional descriptive design	Cross-sectional survey that assessed women’s knowledge of risk factors in pregnancy including exercise, advanced maternal age, folic acid supplementation, infectious disease exposure, medication use, and tobacco use, among other risk factors	Participants included women who presented to an infertility clinic for first physician consultation (*n* = 400)	Knowledge regarding risk of obesity to pregnancy was not evaluated; 90.1% and 82.9% of parous and nulliparous participants strongly believed exercise could affect the outcome of pregnancy; 81.4% and 79.5% of nulliparous participants believed that consumption of certain foods could affect pregnancy; obesity characteristics of the sample were not discussed.	IIIB
Williams [18]	USA	Assess associations between preconception counseling and maternal behaviors before and during pregnancy	Secondary analysis using PRAMS, a national data	Secondary analysis of PRAMS data, which evaluated multiple self-reported behaviors including multivitamin consumption, tobacco and alcohol use, first-trimester prenatal care and compared these variables to receipt of preconception care and counseling	Women with recent live births within 2–6 months of delivery (*n* = 30,481)	Only 32% of participants reported receiving preconception counseling; overweight and obesity was associated with a lower OR of receiving preconception counseling (OR = 0.83, 95% CI 0.74, 0.92; OR = 0.83, 95% CI 0.76, 0.91); 12.4% of participants were overweight and 19.6% were obese.	IIIB
Yamamoto [17]	USA	Evaluate the frequency of preconception counseling about diet and exercise and differences in rates of counseling between women of different weights as well as between different medical specialties	Secondary data analysis using two national survey	Evaluated National Ambulatory Medical Care Survey (NAMCS) and National Hospital Ambulatory Medical Care Survey (NHAMCS) data from office visits. The rates of counseling for diet and exercise were compared between different medical specialties as well as between the weight status of women.	Included both pregnant and non-pregnant women of childbearing age (*n* = 33,187)	Women who were overweight or obese were less likely to receive diet/exercise counseling than non-overweight women (20.0% vs. 26.0%, *p* = 0.05) of available data; 50.2% of visits were with women who were overweight or obese.	IIIB

Note: Quality is indicated by the letter with the level of evidence. For example, IIIB represents the level III and “good” quality. Table presents the alphabetical order of the authors’ name.

## Data Availability

The data presented in this study are available on request to the first or corresponding author.
